# Detection of *bla*_OXA-1_, *bla*_TEM-1_, and Virulence Factors in *E. coli* Isolated From Seals

**DOI:** 10.3389/fvets.2021.583759

**Published:** 2021-03-03

**Authors:** Ana P. Vale, Lynae Shubin, Juliana Cummins, Finola C. Leonard, Gerald Barry

**Affiliations:** ^1^School of Veterinary Medicine, University College Dublin, Dublin, Ireland; ^2^School of Veterinary Medicine, University of California, Davis, Davis, CA, United States; ^3^Central Veterinary Research Laboratory, Backweston Laboratory Complex, Celbridge, Ireland

**Keywords:** antimicrobial resistance, β-lactamases, One Health, seals, virulence factors, *E. coli*

## Abstract

Marine mammals are frequently considered good sentinels for human, animal and environmental health due to their long lifespan, coastal habitat, and characteristics as top chain predators. Using a One Health approach, marine mammals can provide information that helps to enhance the understanding of the health of the marine and coastal environment. Antimicrobial resistance (AMR) is the quintessential One Health problem that poses a well-recognised threat to human, animal, and ecosystem health worldwide. Treated and untreated sewage, hospital waste and agricultural run-off are often responsible for the spread of AMR in marine and freshwater ecosystems. Rescued seals (*n* = 25) were used as sentinels to investigate the levels of AMR in the Irish coastal ecosystem. Faecal swabs were collected from these animals and bacterial isolates (*E. coli* and cefotaxime-resistant non-*E. coli*) from each swab were selected for further investigation. *E. coli* isolates were characterised in terms of phylogenetic group typing, AMR, and virulence factors. All *E. coli* isolates investigated in this study (*n* = 39) were ampicillin resistant while 26 (66.6%) were multi-drug resistant (MDR). Resistance genes *bla*_OXA−1_ and *bla*_TEM−1_ were detected in 16/39 and 6/39 isolates, respectively. Additionally, virulence factors associated with adhesion (*sfa, papA*, and *papC*) and siderophores (*fyuA* and *iutA*) were identified. An additional 19 faecal cefotaxime-resistant non-*E. coli* isolates were investigated for the presence of β-lactamase encoding genes. These isolates were identified as presumptive *Leclercia, Pantoea* and *Enterobacter*, however, none were positive for the presence of the genes investigated. To the authors knowledge this is the first study reporting the detection of *bla*_OXA−1_ and *bla*_TEM−1_ in phocid faecal *E. coli* in Europe. These results highlight the importance of marine mammals as sentinels for the presence and spread of AMR in the marine and coastal environment.

## Introduction

Located in the North Atlantic region, Ireland offers an important habitat for marine mammals including harbour seals (*Phoca vitulina*) and grey seals (*Halichoerus grypus*) in search of haul-out sites during breeding and moulting (1). Grey seals are known to migrate between countries, but harbour seals tend to travel less widely. Nonetheless, both species usually return to their breeding areas (2).

Marine mammals are frequently considered good sentinels for human and environmental health because their position at the top of the food chain, their long-life span and their coastal habitat can provide an early warning system for public health issues (3, 4). Using a One Health approach, marine mammals can be seen as an important source of information that helps to enhance our understanding of the health of the marine and coastal environment (3).

Antimicrobial resistance (AMR) is the quintessential One Health problem (5) that poses a well-recognised threat to human, animal and ecosystem health worldwide (6). Much of this problem has been associated with the misuse of antimicrobials in human, veterinary and agricultural settings (7) leading to the increased emergence of antimicrobial-resistant bacteria (ARB) in marine and fresh water ecosystems (8). Treated and untreated sewage, hospital waste and agricultural run-off are often responsible for the spread of AMR in these ecosystems (9–11). Studies have shown that natural environments, such as soils, sediments, and surface waters have complex microbiomes which include clinically important ARBs and antimicrobial-resistant genes (ARGs) (12). ARGs can be transferred into soils and leached to groundwater or carried by runoff and erosion to surface water (13). In addition, dense bacterial populations in treatment plants facilitate frequent genetic exchange through mobile genetic elements (MGE), such as plasmids, integrons, and transposons (8, 14). For example, the spread of β-lactamases, enzymes responsible for decreasing the efficacy of critically important β-lactam antimicrobials against Gram negative bacteria, is frequently due to MGEs (15). These enzymes are currently the most important mechanism of resistance in Gram negative pathogens with more than 2,600 enzymes described to date. The most frequently described enzymes in *E. coli* include CTX-M, TEM and SHV, Ambler class A enzymes, and OXA, Ambler class D enzymes (16). Also disseminated by MGEs, virulence factors including adherence factors, invasion factors, iron acquisition systems, capsules and toxins facilitate bacterial colonisation of the host (17).

An Irish technical report identified high levels of resistant *E. coli* in urban wastewater (18). More recently, Mahon et al. were the first in Europe to report the isolation of New Delhi metallo-beta-lactamase (NDM)-producing *Enterobacteriaceae* from both fresh water and seawater sampled on 2 Irish beaches located near an untreated human sewage ocean discharge (19). This finding raises concerns regarding the potential of sewage discharges to contribute to the spread of ARBs and ARGs in the environment, especially when recent studies have shown that resistant bacteria can be selected at extremely low antibiotic concentrations, similar to concentrations found in some aquatic and soil environments (20, 21). Although it is recognised that proximity to human activities shapes the AMR profile of the gastrointestinal microbiome of wild mammals, the presence of ARBs and ARGs in the intestinal microbiota of wild animals has not been thoroughly investigated (8, 22, 23). Additionally, the role played by wildlife colonised with ARB in the dissemination of ARGs worldwide needs to be addressed (24, 25).

In light of the recent findings of ARB in Irish coastal waters and the complexity of the factors governing dissemination of AMR in the environment, a pilot study was conducted to characterise the faecal *E. coli* populations of pinnipeds living in coastal waters surrounding Ireland, and investigate the presence of β-lactamase encoding genes and virulence factors. Furthermore, the presence of β-lactamase encoding genes was examined in cefotaxime-resistant non-*E. coli* isolates.

## Materials and Methods

### Animals

In the summer breeding season of 2017, collection of faecal swabs from 23 harbour seals (*P. vitulina*) and two grey seals (*H. grypus*) was attempted at the premises of Seal Rescue Ireland (SRI), the only marine rehabilitation centre in the Republic of Ireland; however, three animals did not defecate during the visit. Convenience sampling was conducted on two occasions in July 2017, 22 days apart, to sample as many individual animals as possible (Table 1). Fourteen and eleven animals were sampled on the first and second sampling-days, respectively, which made up the total number of animals housed at SRI at the time (Table 1).

**Table 1:** Sampling details including animal identification, sampling day and bacteria isolated from faeces; *E. coli* isolated from TBX supplemented with cefotaxime (REC), *E. coli* isolated from TBX (EC) and non- *E. coli* isolated from TBX supplemented with cefotaxime (RC) according to sampling-day (3rd of July and 25th of July).

**Seal** ** ID**	**Isolate ID**	**Sampling** ** day**	**Bacterial** ** species**	**Phylogenetic** ** group** ** typing**	**Resistance** ** phenotype**	**Resistance** ** genotype**	**Virulence** ** factors**	**Previous** ** antibiotic** ** treatment**	**No. days** ** between** ** treatment** ** and sampling**
1	1EC1	1	*E. coli*	B1	AMP, PIP, ENR, TET		*sfa*	N.A.	N.A.
	1EC2	1	*E. coli*					N.A.	N.A.
2	2EC1	1	*E. coli*	B1	AMP			N.A.	N.A.
	2EC2	1	*E. coli*					N.A.	N.A.
3	3EC1	1	*E. coli*					N.A.	N.A.
	3EC2	1	*E. coli*	B1	AMP, PIP, ENR, TET	*bla*_TEM−1_		Marbofloxacin	39
4	4EC1	1	*E. coli*	B1	AMP, CHL			N.A.	N.A.
	4EC2	1	*E. coli*					N.A.	N.A.
5	5EC1	1	*E. coli*	B1	AMP, CHL			N.A.	N.A.
	5EC2	1	*E. coli*					N.A.	N.A.
6	6EC1	2	*E. coli*	B1	AMP, PIP, TOB, SXT	*bla*_TEM−1_	*fyuA, iutA, sfa, papC*	N.A.	N.A
	6EC2	1	*E. coli*	B1	AMP, PIP, TOB, STX	*bla*_TEM−1_	*fyuA, iutA, papA*	N.A	N.A.
	6EC3	1	*E. coli*	B1	AMP, CEV, CHL			N.A.	N.A.
7	7EC1	1	*E. coli*	B1	AMP, CHL			N.A.	N.A.
	7EC2							N.A.	N.A.
9	9EC1	1	*E. coli*	A/C^a^	AMP			N.A.	N.A.
	9EC2	1	*E. coli*	B1	AMP, PIP, ENR, TET	*bla*_TEM−1_		N.A.	N.A.
10	10EC1	1	*E. coli*	A	AMP			N.A.	N.A.
	10EC2	1	*E. coli*	B1	AMP			N.A.	N.A.
13	13EC1	1	*E. coli*	B1	AMP, PIP, ENR, TET	*bla*_TEM−1_		N.A.	N.A.
	13EC2								
14	14EC1	1	*E. coli*	B2	AMP			N.A.	N.A.
	14EC2								
12	12REC1	2	*E. coli*	A/C^a^	AMP, AMC, PIP, CEF, GEN, TOB, ENR, MAR, CHL, STX	*bla*_OXA−1_	*fyuA, iutA, sfa, papC*	N.A.	N.A.
	12REC2								
16	16REC1	2	*E. coli*	A/C^a^	AMP, AMC, PIP, CEF, GEN, TOB, ENR, MAR, CHL, STX	*bla*_OXA−1_	*fyuA, iutA, papA*	Enrofloxacin Amoxicillin/ Clavulanate	202 192
	16REC2	2	*E. coli*	A/C^a^	AMP, AMC, PIP, CEF, AMK, GEN, TOB, ENR, MAR, CHL, STX	*bla*_OXA−1_	*fyuA, iutA, papA*		
17	17REC1	2	*E. coli*	A/C^a^	AMP, AMC, PIP, LEX, CPD, CEF, GEN, TOB, ENR, MAR, CHL, STX	*bla*_OXA−1_	*fyuA, iutA, sfa, papC, papA*	N.A.	N.A.
	17REC2	2	*E. coli*	A/C^a^	AMP, AMC, PIP, LEX, CPD, CEF, GEN, TOB, ENR, MAR, CHL, STX	*bla*_OXA−1_	*fyuA, iutA, papC, papA*		
18	18REC1	2	*E. coli*	A/C^a^	AMP, AMC, PIP, CEF, GEN, TOB, ENR, MAR, CHL, STX	*bla*_OXA−1_	*fyuA, papA*	Marbofloxacin	0
	18REC2	2	*E. coli*	A/C^a^	AMP, AMC, PIP, GEN, TOB, ENR, MAR, CHL, STX	*bla*_OXA−1_	*fyuA, papA*		
19	19REC1	2	*E. coli*	A/C^a^	AMP, AMC, PIP, CEF, GEN, TOB, ENR, MAR, CHL, STX	*bla*_OXA−1_	*fyuA, iutA, papC, papA*	Marbofloxacin	1
	19REC2								
20	20REC1	2	*E. coli*	A/C^a^	AMP, AMC, PIP, CEF, GEN, TOB, ENR, MAR, CHL, STX	*bla*_OXA−1_	*fyuA, iutA, papC, papA*	Marbofloxacin	1
	20REC2								
23	23REC1	2	*E. coli*	A/C^a^	AMP, AMC, PIP, CEF, GEN, TOB, ENR, MAR, CHL, STX	*bla*_OXA−1_	*fyuA, iutA, papC, papA*	N.A.	N.A.
	23REC2								
24/25	24/25REC1	2	*E. coli*	A/C^a^	AMP, AMC, PIP, CEF, ENR, MAR, CHL, STX	*bla*_OXA−1_	*fyuA, iutA, papC, papA*	Marbofloxacin	2
	24/25REC2	2	*E. coli*	A/C^a^	AMP, AMC, PIP, CEF, GEN, TOB, ENR, MAR, CHL, STX	*bla*_OXA−1_	*fyuA, iutA, papC, papA*		
1–10 and 13–14	1RC1 1RC2 2RC1 2RC2 3RC1 3RC2 4RC2 5RC1 5RC2 6RC1 7RC1 7RC2 8RC1 8RC2 9RC2 10RC1 13RC2 13RC3 14RC3	1	Non-*E. coli*	N.A.	N.A.		N.A.	N.A.	N.A.

*Characterisation of faecal E. coli isolated from seals according to their phylogenetic group typing, virulence factors, antimicrobial resistance genes (genotype), and antimicrobial susceptibility profile (phenotype) given by Vitek2. For the purpose of this study intermediate antimicrobial susceptibility was interpreted as resistant. Antimicrobials tested: AMP, Ampicillin; AMC, Amoxicillin/Clavulanic Acid; PIP, Piperacillin; LEX, Cefalexin; CPD, Cefpodoxime; CEV, Cefovecin; CEF, Ceftiofur; IPM, Imipenem; AMK, Amikacin; GEN, Gentamicin; TOB, Tobramycin; ENR, Enrofloxacin; MAR, Marbofloxacin; TET, Tetracycline; NIT, Nitrofurantoin; CHL, Chloramphenicol; PMB, Polymyxin B; SXT, Trimethoprim/Sulfamethoxazole. Additional information on antimicrobial use, number of days between antimicrobial treatment and sampling-time for the samples with antimicrobial resistance genes. N.A., not applicable*.

a *Phylogenetic groups A and C could not be differentiated*.

Sterile cotton swabs were used to collect freshly voided faeces from each animal's enclosure (individual pens with covered roof) without contacting the floor. Enclosures at SRI are cleaned daily; thorough washing and disinfection with bleach and Virkon^©^ are performed before any new animal is moved into an enclosure.

Faecal swabs were kept refrigerated for a period no longer than 24 h before being processed in the laboratory.

At time of sampling, the age of animals ranged from 9 days to 10 months approximately. Samples were collected from animals between 24 h and 8 months after their arrival at the SRI facilities.

### Faecal Swab Processing and *E. coli* Isolation

In the microbiology laboratory, the faecal swabs were placed into 20 mL sterile plastic tubes filled with 5 mL of buffered peptone water (BPW, Lab M) and vortexed for 10 s.

Aliquots of 0.1 mL of each initial suspension were plated onto the chromogenic selective medium Tryptone Bile X-glucuronide (TBX, Fisher Scientific) and TBX supplemented with cefotaxime (sc-202989 Cefotaxime Sodium Salt; 0.250 mg/L according to EUCAST epidemiological cut-off value (ECOFF) at the time of the study). TBX and TBX supplemented with cefotaxime were used to detect cefotaxime-susceptible and cefotaxime-resistant *E. coli* colonies (blue/green colonies), respectively. Sterile spreaders were used to evenly distribute the faecal suspension across the plates and then all plates were incubated at 37°C for a period of 20–24 h. Two to three colonies were isolated from each plate/sample if colonies differed phenotypically. *E. coli* ATCC 25922 and extended spectrum β-lactamase (ESBL)-producing isolate R5S (26) were used as negative and positive controls, respectively.

### Antimicrobial Susceptibility Testing of *E. coli*

Thirty-nine *E. coli* isolates (selected from TBX and TBX supplemented with cefotaxime media) were grown on blood agar plates for 18 h at 37°C before testing for antimicrobial susceptibility by the VITEK 2 automated system (Biomerieux^©^), as recommended by the manufacturer (Table 1). Vitek 2 AST-GN65 cards (Biomerieux^©^) were used to investigate the susceptibility of the isolates to amoxicillin, ampicillin, amoxicillin and clavulanic acid, piperacillin, cefalotin, cefalexin, cefpodoxime, cefovecin, ceftiofur, imipenem, amikacin, gentamicin, tobramycin, enrofloxacin, marbofloxacin, tetracycline, nitrofurantoin, chloramphenicol, and trimethoprim/sulfamethoxazole. Results were interpreted according to the Clinical and Laboratory Standards Institute (CLSI) guidelines. *E. coli* ATCC 25922 was used as control strain (27–31).

Rapid DNA extraction was performed on all isolates by the boiling method (32).

### Investigation of Phylogenetic Group Typing and Virulence Factors of *E. coli*

The phylogenetic group of 33 *E. coli* was investigated using an adapted version of the Clermont method (Table 2) (33). Each isolate was assigned to a group (A, B1, B2, C, D, E, F) according to the presence or absence of genes *arpA, chuA, yjaA*, and the DNA fragment TSPE4.C2 (33). Positive controls were provided by the Galway University Hospital National Microbiology Reference Laboratory.

**Table 2:** List of primers, target genes, primer sequences, annealing temperatures, and primer concentrations used for *E. coli* phylogenetic group typing in this study.

**Primer ID**	**Target**	**Primer sequence (5**^**′**^- 3^**′**^**)**	**Annealing T (^**°**^C)**	**Final concentration (μM)**	**PCR product (bp)**	**PCR reaction**	**References**
chuA.1b	*chuA*	ATGGTACCGGACGAACCAAC	60	0.3	288	Quadruplex	(a)
chuA.2		TGCCGCCAGTACCAAAGACA		0.3			
yjaA.1b	*yjaA*	CAAACGTGAAGTGTCAGGAG		0.6	211		
yjaA.2b		AATGCGTTCCTCAACCTGTG		0.6			
TspE4C2.1b	TspE4.C2	CACTATTCGTAAGGTCATCC		0.7	152		
TspE4C2.2b		AGTTTATCGCTGCGGGTCGC		0.7			
AceK.f	*arpA*	AACGCTATTCGCCAGCTTGC		0.3	400		
ArpA1.r		TCTCCCCATACCGTACGCTA		0.3			
trpAgpC.1	*trpA*	AGTTTTATGCCCAGTGCGAG	59	0.2	219	Group C duplex	(b)
trpAgpC.2		TCTGCGCCGGTCACGCCC		0.2			
ArpAgpE.f	*arpA*	GATTCCATCTTGTCAAAATATGCC	57	0.2	301	Group E duplex	
ArpAgpE.r		GAAAAGAAAAAGAATTCCCAAGAG		0.2			
trpBA.f	*trpA*	CGGCGATAAAGACATCTTCAC	59/57	0.2	489	Internal control group C and E	(c)
trpBA.r		GCAACGCGGCCTGGCGGAAG		0.2			

*Adapted from (a) Clermont et al. (33) and Tim Julian (Eawag, Switzerland), (b) Lescat et al. (34), and (c) Clermont et al. (35)*.

Briefly, all PCR reactions were performed in a final volume of 25 μl containing 1× master mix [2× Qiagen Multiplex PCR Master Mix, final primer concentrations of 0.2–0.7 μM as appropriate (Table 2), PCR grade water] and 1.5 μl of bacterial lysate. PCR reactions were performed as follows: denaturation 15 min at 95°C, followed by 30 cycles of 20 s at 94°C, 20 s at 60°C, and 30 s at 72°C with a final extension step of 5 min at 72°C. PCR products were loaded on 2% agarose gels with SYBR^®^ Safe DNA gel stain and run for 60 min at 100 V. DNA bands were visualised using a UV-transilluminator. For groups C and E, two further PCRs were performed using the previous protocol with 5× Q-Solution (Qiagen^©^) included in the master mix. PCR reactions were performed as follows: denaturation 15 min at 95°C, 30 cycles of 20 s at 94°C, 20 s at 59°C (group C) or 57°C (group E), respectively and 30 s at 72°C, with a final extension step of 5 min at 72°C.

Selected genes encoding virulence factors associated with adhesion (*afaE8, papA, papC*, and *sfa*), capsular antigen (*kpsMFII*), toxins (*CNF1*), and siderophores (*fyuA* and *iutA*) were also investigated as previously published (36–40). Positive PCR products were Sanger sequenced for identification of gene variants. The nucleotide sequence queries were loaded into the Virulence Factor Database (VFDB) (41).

### Identification of Cefotaxime-Resistant Non-*E. coli*

Faecal samples collected on the first-sampling day did not yield any *E. coli* colonies that grew on medium supplemented with cefotaxime. However, 19 other colonies that were not *E. coli* were selected from this medium for further analysis (Table 1). Identification of these colonies was carried out by performing 16s rRNA PCR according to Marchesi et al. (42). PCR products (1,300 bp) were Sanger sequenced and the nucleotide sequence queries were loaded into the National Centre for Biotechnology Information (NCBI) Basic Local Alignment Search Tool (BLAST^©^). The highest query cover, identity and max score were used to determine the best fit for sequence alignment.

### Investigation of β-Lactamase-Encoding Genes

Thirty-three *E. coli* isolates yielding a phenotype of resistance to ampicillin (Table 1) were tested to see if they contained β-lactamase-coding genes SHV, TEM, and OXA (Multiplex I) while 16 isolates with susceptibility reported as intermediate or resistant to 3rd generation cephalosporins (Table 1) were further investigated for the presence of ESBL (CTX-M) (Multiplex II) and plasmid-mediated AmpC (ACC, FOX, MOX, CMY, DHA, LAT, ACT, BIL, MIR) (Multiplex III) encoding genes (32). Additionally, 19 cefotaxime-resistant non-*E. coli* isolates were investigated for the presence of β-lactamases. Positive controls were provided by the Galway University Hospital National Microbiology Reference Laboratory.

Briefly, for multiplex PCRs I, II and III, reactions were carried out in 25 μl of reaction mix containing master mix (2× Qiagen Multiplex PCR Master Mix, 5× Q-Solution (Qiagen), primers at concentration of 0.2–0.5 μM as appropriate, PCR grade water) and 1.0 μl of bacterial lysate. PCR reactions were performed as follows: denaturation 15 min at 95°C, 30 cycles of 30 s at 94°C, 90 s at 60°C, and 90 s at 72°C with a final extension step of 10 min at 72°C.

Agarose gels ranging between 1.2 and 2% (according to the size of PCR product) were run at 100 V for 60 min. A UV-transilluminator was used to visualise the PCR products.

Positive PCR products were Sanger sequenced. The nucleotide sequence queries were loaded into the Comprehensive Antibiotic Resistance Database (CARD) (43).

## Results

### Cefotaxime Resistant *E. coli*

Faecal samples from 22 seals were collected over two sampling days. Samples were plated onto TBX and TBX supplemented with cefotaxime. After overnight incubation, samples were examined for growth and the results are shown in Table 1. *E. coli* were not retrieved from faeces sampled from 2 animals, while there was no *E. coli* growth on TBX supplemented with cefotaxime on samples collected on day 1. *E. coli* was recovered from all faecal samples from day 2 plated on TBX supplemented with cefotaxime.

### Antimicrobial Susceptibility Testing of *E. coli*

In total, 39 *E. coli* isolates were investigated in the present study; 23 from the faeces collected on sampling-day 1 and 16 from sampling-day 2. All isolates were ampicillin-resistant, 16 of them were also resistant to amoxicillin and clavulanic acid (Table 1), while 22 of the isolates were intermediately susceptible or resistant to fluoroquinolones. From day 1 *E. coli* isolates, 10 (43.5%) were multidrug resistant (MDR) showing resistance to 3–4 different antimicrobial classes (penicillins, cephalosporins, tetracyclines, and potentiated sulphonamides) (44). In contrast, all *E. coli* isolates from sampling-day 2 were MDR, displaying resistance to 4–6 different antimicrobial classes including penicillins, fluoroquinolones, amphenicols, and potentiated sulphonamides (Table 1).

### Molecular Investigation of *E. coli*

From a total of 39 *E. coli* isolates, 33 were selected for analysis by PCR and sequencing. Twenty-two carried β-lactamase encoding genes; *bla*_TEM−1_ was detected in six *E. coli* isolated from four seals on Day 1 while *bla*_OXA−1_ was detected in 16 *E. coli* isolated from nine animals on Day 2. Of four seals shedding TEM-1 *E. coli*, two originated from county Galway and one had been treated at SRI with marbofloxacin, 39 days before sampling. Of nine seals shedding *E. coli* carrying *bla*_OXA−1_, five had been medicated with marbofloxacin (Table 1). Reasons for medication included wounds, otitis, and umbilical abscess.

Additionally, the presence of CTX-M and AmpC encoding genes was investigated in the 16 isolates from day 2 (cefotaxime-resistant *E. coli*) using multiplex II and multiplex III PCRs; however, none of these genes was detected.

*E. coli* isolates belonged to phylogenetic groups A (*n* = 1), B1 (*n* = 13), B2 (*n* = 2), and A/C (*n* = 17) with the all 16 isolates from Day 2 belonging to group A/C. For isolates characterised as A/C it was not possible to further determine their phylogenetic group.

To further characterise the *E. coli* isolates investigation of selected virulence factors was performed. From day 1 samples, 3 of 17 *E. coli* isolates carried at least one virulence factor associated with adhesion (*sfa, papA*, and *papC*) and/or siderophores (*fyuA* and *iutA*) while all *E. coli* (16) isolated on the second sampling day carried at least two virulence factors. Some isolates carried multiple virulence factors including isolate 17REC1 that carried five of the eight virulence factors investigated.

### Investigation of Non-*E. coli*

Cefotaxime-resistant non-*E. coli* isolates were grown from samples collected on both sampling-days and 19 colonies recovered on the first sampling-day were selected for further investigation based on colony morphology.

16s rRNA PCR was used to amplify a specific region of the genome of each isolate. Despite the limitations of this method, sequence homology suggests that most of the isolates belong to the genera *Leclercia, Enterobacter, Pantoea*, and/or *Psychrobacter* (Supplementary File). Definitive species identification was not established. None of these isolates carried any of the β-lactamase encoding genes investigated (Table 1).

## Discussion

To the authors' knowledge this is the first study reporting the detection of *bla*_OXA−1_ and *bla*_TEM−1_ in phocid faecal *E. coli* in Europe. The presence of β-lactamase producing *E. coli* in the microbiota of wild seals, some of which had not been previously medicated with antimicrobials is a cause for concern and highlights their potential to serve as One Health sentinels when investigating AMR. There is also scope to explore zoonotic diseases including avian influenza and environmental contamination by heavy metals and domoic acid, among other things, using these species as sentinels.

All 39 *E. coli* isolated from seal faeces at the SRI marine rehabilitation centre were ampicillin resistant and 26 of 39 (66.6%) were MDR, which highlights the presence of MDR bacteria in the microbiome of marine mammals. Despite rigorous cleaning and disinfection protocols at the SRI centre, it cannot be proven that faecal isolates were not simply representative of the in-house flora of the centre however, the isolation of MDR *E. coli* from animals only recently arrived in the rescue centre strongly suggests that the organisms may have been present in the gastrointestinal tract of at least some of the seals before arrival at the centre. It is noteworthy that all *E. coli* (16) isolated on the second sampling-day were MDR which contrasts with 10 MDR *E. coli* of 23 *E. coli* recovered on the first sampling-day. Interestingly, the number of MDR *E. coli* isolates sampled on these two sampling-days differs considerably. The only major difference recorded between the 2 days was an outbreak of disease due to phocid herpes virus diagnosed soon after the second sampling-day. Whether this bears any relationship with the findings of this study is unclear. Research has shown that neurohormones released in the gut as a result of stressful events can increase the rate of horizontal gene transfer of genes encoding for AMR which can lead to an increase in shedding of resistant bacteria (45–47). Although little is known about the impact of acute viral infections on the composition and kinetics of the microbiome, these types of infections could be classified as systemic stressful events and therefore one could hypothesise that an increase in horizontal gene transfer may occur (48, 49). Fifty-six per cent of *E. coli* investigated showed resistance to fluoroquinolones, which may be associated with the use of marbofloxacin at the SRI, although not all isolates with resistance to fluoroquinolones came from animals with a history of marbofloxacin treatment.

In Ireland, a wide range of β-lactamases has been reported in bacteria isolated from humans, companion animals, production animals and wastewaters (26, 50–54). Karczmarczyk et al. (52) identified *bla*_TEM_ in 89.2% of the *E. coli* examined in their study while *bla*_OXA_ were detected in 1.35%. Additionally, Carroll et al. (55) identified *bla*_TEM_ in faecal *E. coli* sampled from one Irish herring gull and one Irish black-headed gull while another study identified *bla*_CTX−M_ group 1 in 4.5% of the samples collected from Irish gulls (55, 56). In line with this, isolates with either *bla*_OXA_ or *bla*_TEM_ were identified in the faecal samples collected, suggesting these genes are circulating in the marine environment also. ESBL-producing *E. coli* have been identified in more than 30 wild animal species (11) but none were identified in the *E. coli* or non-*E. coli* isolates investigated in this study. While this does not rule out their presence, it may suggest that these genes are not as prevalent in the environment of these seals as the ones that were identified.

This study further characterised *E. coli* isolated from seals in terms of phylogenetic groups and virulence factors. Due to the lack of information in the literature, a subset of virulence factors was selected for investigation, based on data available for *E. coli* isolated from domestic animals. In this study, virulence factors associated with adhesion and siderophores were detected in many isolates. Despite the constraints of the small number of animals investigated and further bias by the selection criteria of *E. coli* (1, 2, or 3 colonies per sample according to colony phenotype), a difference between the number of *E. coli* carrying virulence genes on each sampling day is clear. Horizontal gene transfer is an essential mechanim for the spread of virulence determinants between different bacterial strains and species (57). Moreover, studies have shown that stress can induce the release of norepinephrine in the gut and this catecholamine can promote horizontal gene transfer by conjugation and influence the production of virulence factors including toxins and adhesins in *E. coli* (46, 58–61). It is possible that the differences reported between sampling day 1 and day 2 were triggered by the herpes virus infection that was subsequently diagnosed (48, 62).

Studies have shown that ecological niches and life events impact the phylogenetic group dynamics and diversity of *E. coli* (63, 64). In the present study, phylogenetic groups A, B1, B2, and A/C were detected on the first sampling day while on the second sampling day only A/C *E. coli* were detected. Further characterisation of A/C isolates was not possible due to non-specific DNA amplification. Differences in the antimicrobial susceptibility profile and virulence factors exclude the possibility of clonal spread of A/C *E. coli* on the second sampling day. These suprising findings, including the number of MDR isolates, number of *E. coli* carrying β-lactamases and virulence factors and phylogenetic diversity detected on two different sampling days suggest differences in the population sampled on these two occasions again pointing to the impact a natural herpesvirus infection could have had on the profile of the samples. These data highlight the importance of examining the resistome of sentinal species throughout time.

Phocid faecal cefotaxime-resistant non-*E. coli* isolates were homologous to members of the *Enterobacterales: Leclercia, Pantoea*, and *Enterobacter*. *Leclercia adecarboxylata* is an opportunistic pathogen associated with water affecting both immunocompromised and immunocompetent patients (65). Studies have reported *Leclercia adecarboxylata* susceptibility to cephalosporins and *bla*_SHV−12_ has been identified in *Leclercia adecarboxylata* clinical samples (66, 67). *Pantoea agglomerans* may cause infections in humans and is variably susceptible to antimicrobials while *Enterobacter ludwigii*, previously included in the *Enterobacter cloacae* complex, is a MDR bacterium that can carry β-lactamase encoding genes (68–71). Because all the above bacteria belong to the *Enterobacterales* order, distinction between species is complex. For more accurate identification, PCR protocols investigating genetic characteristics other than 16S rRNA would be required.

Despite the fact that animals sampled in this study represented all live stranded seals in Ireland housed at SRI during the period of the trial, the relatively small sample size is a limitation of this study. A larger population would have given a better idea of the magnitude of the problem, but it is clear that even with a small sample size, this study has pinpointed issues and has provided justification and a roadmap for future studies in this area. Stringent cleaning and disinfection and other infection control protocols in place at SRI and a rigorous sampling technique greatly reduced the possibility of cross-contamination between different enclosures/pens.

It is difficult to determine the exact origin of β-lactamase encoding genes circulating in the population of young Irish seals as they share their costal habitat with different species, but the fact that *bla*_TEM−1_ and b*la*_OXA−1_ were present in their faeces and MDR *E. coli* were frequently detected, is concerning. The presence of β-lactamases jeopardises the use of critically important antimicrobials including penicillins and cephalosporins and the findings of this study indicate the spread of AMR mechanisms to bacteria in coastal areas.

Marine mammals can act as reservoirs, vectors, and bioindicators of resistant bacteria and AMR genes in the environment (72, 73). Treated and untreated sewage, hospital waste, aquaculture discharges and agricultural runoff provide means to deliver antibiotics, pollutants and resistant bacteria to the aquatic environment, thus playing a major role in driving ARG transfer, ecology, and evolution (14). Additionally, seals can interact with other marine wildlife and birds and engage in the transfer of ARB between populations across large parts of the world. Future investigations should acknowledge the presence of these issues and seek to understand the movement of ARGs between populations and the extent to which global spread of ARGs in human populations is reflected in wild animal populations.

This study shows that some isolates of *E. coli* carried β-lactamase encoding genes (*bla*_OXA−1_ or *bla*_TEM−1_) as well as virulence factors associated with adhesion (*sfa, papA*, and *papC*) and/or siderophores (*fyuA* and *iutA*). While harbour seals have the potential to migrate to different locations, they tend to return to the same breeding grounds (2) and young pups similar to the ones sampled in this study do not tend to migrate long distances. The presence of MDR bacteria in the seal pups indicates that were probably acquired locally, however, it is also possible that the adult seals or other migratory wildlife in the area may have acquired these resistant bacteria elsewhere, brought them to Ireland and passed them to the pups. At this point, although it is difficult to identify the geographic source exactly, the data presented in this study clearly establishes the presence of MDR *E. coli* circulating in the Irish marine environment at the time of sampling.

## Data Availability Statement

The original contributions presented in the study are included in the article/supplementary material, further inquiries can be directed to the corresponding author/s.

## Ethics Statement

Ethical review and approval was not required for the animal study because the seals were not manipulated for the purpose of collecting faecal swabs as these were sampled from the floor. This study was part of a bigger project that got exemption from full ethical review according to the University College Dublin Animal Research Ethics Committee (AREC-E-17-24-Barry).

## Author Contributions

AV designed the study, conducted the experiments, and wrote the manuscript. LS and JC designed the study and conducted the experiments. FL and GB designed the study and critically reviewed the manuscript. All authors contributed to the manuscript and approved the submitted version.

## Conflict of Interest

The authors declare that the research was conducted in the absence of any commercial or financial relationships that could be construed as a potential conflict of interest.

## References

[1] Cronin MA. The conservation of seals in Irish waters: how research informs policy. Mar Policy. (2011) 35:748–55. 10.1016/j.marpol.2011.01.006

[2] Bonner W. The grey and common seal in European waters. Oceangr Mar Biol Ann Rev. (1972) 10:461–507.

[3] Bossart GD. Marine mammals as sentinel species for oceans and human health. Vet Pathol. (2011) 48:676–90. 10.1177/0300985810388525 21160025

[4] Reif JS. Animal sentinels for environmental and public health. Public Health Rep. (2011) 126:50–7. 10.1177/00333549111260S108 21563712 PMC3072903

[5] Robinson TP Bu DP Carrique-Mas J Fèvre EM Gilbert M Grace D. Antibiotic resistance is the quintessential One Health issue. Trans R Soc Trop Med Hyg. (2016) 110:377–80. 10.1093/trstmh/trw048 27475987 PMC4975175

[6] WHO, FAO, OIE. The FAO-OIE-WHO Collaboration Sharing Responsibilities and Coordinating Global Activities to Address Health Risks at the Animal-Human-Ecosystems Interfaces. WHO, FAO, OIE (2010).

[7] OIE. OIE List of Antimicrobial Agents of Veterinary Importance. OIE (2015).

[8] Allen HK Donato J Wang HH Cloud-Hansen KA Davies J Handelsman J. Call of the wild: antibiotic resistance genes in natural environments. Nat Rev Microbiol. (2010) 8:251–9. 10.1038/nrmicro2312 20190823

[9] Baquero F Martínez JL Cantón R. Antibiotics and antibiotic resistance in water environments. Curr Opin Biotechnol. (2008) 19:260–5. 10.1016/j.copbio.2008.05.006 18534838

[10] Taylor NGH Verner-Jeffreys DW Baker-Austin C. Aquatic systems: maintaining, mixing and mobilising antimicrobial resistance? Trends Ecol Evol. (2011) 26:278–84. 10.1016/j.tree.2011.03.004 21458879

[11] Wellington EMH Boxall ABA Cross P Feil EJ Gaze WH Hawkey PM. The role of the natural environment in the emergence of antibiotic resistance in Gram-negative bacteria. Lancet Infect Dis. (2013) 13:155–65. 10.1016/S1473-3099(12)70317-1 23347633

[12] Leonard AFC Zhang L Balfour AJ Garside R Gaze WH. Human recreational exposure to antibiotic resistant bacteria in coastal bathing waters. Environ Int. (2015) 82:92–100. 10.1016/j.envint.2015.02.013 25832996

[13] Zhang XX Zhang T Fang HHP. Antibiotic resistance genes in water environment. Appl Microbiol Biotechnol. (2009) 82:397–414. 10.1007/s00253-008-1829-z 19130050

[14] Marti E Variatza E Balcazar JL. The role of aquatic ecosystems as reservoirs of antibiotic resistance. Trends Microbiol. (2014) 22:36–41. 10.1016/j.tim.2013.11.001 24289955

[15] Li XZ Mehrotra M Ghimire S Adewoye L. β-Lactam resistance and β-lactamases in bacteria of animal origin. Vet Microbiol. (2007) 121:197–214. 10.1016/j.vetmic.2007.01.015 17306475

[16] De Oliveira D Forde B Kidd T Harris P Schembri M Beatson S. Antimicrobial resistance in ESKAPE pathogens. Clin Microbiol Rev. (2020) 33:e00181-19. 10.1128/CMR.00181-19 32404435 PMC7227449

[17] Daga A Koga V Soncini J Matos C Marcia P Pelisson M. *Escherichia coli* bloodstream infections in patients at a University hospital: virulence factors and clinical characteristics. Front Cell Infect Microbiol. (2019) 9:191. 10.3389/fcimb.2019.00191 31245301 PMC6563721

[18] Morris D Harris S Morris C Commins E Cormican M. Hospital Effluent: Impact on the Microbial Environment and Risk to Human Health. Dublin: EPA (2015).

[19] Mahon BM Brehony C McGrath E Killeen J Cormican M Hickey P. Indistinguishable NDM-producing *Escherichia coli* isolated from recreational waters, sewage, and a clinical specimen in Ireland, 2016 to 2017. Eurosurveillance. (2017) 22:1–5. 10.2807/1560-7917.ES.2017.22.15.30513 28449738 PMC5476983

[20] Kümmerer K. Antibiotics in the aquatic environment–a review–part, I. Chemosphere. (2009) 75:417–34. 10.1016/j.chemosphere.2008.11.086 19185900

[21] Gullberg E Cao S Berg OG Ilbäck C Sandegren L Hughes D. Selection of resistant bacteria at very low antibiotic concentrations. PLoS Pathog. (2011) 7:e1002158. 10.1371/journal.ppat.1002158 21811410 PMC3141051

[22] Bruno C Albino B François-Xavier M. Wildlife, exotic pets, and emerging zoonoses. Emerg Infect Dis. (2009) 13:1–7. 10.3201/eid1301.060480 17370509 PMC2725831

[23] Greig J Rajić A Young I Mascarenhas M Waddell L LeJeune J. A scoping review of the role of wildlife in the transmission of bacterial pathogens and antimicrobial resistance to the food chain. Zoonoses Public Health. (2015) 62:269–84. 10.1111/zph.12147 25175882

[24] Smet A Martel A Persoons D Dewulf J Heyndrickx M Herman L. Broad-spectrum β-lactamases among Enterobacteriaceae of animal origin: molecular aspects, mobility and impact on public health. FEMS Microbiol Rev. (2009) 34:295–316. 10.1111/j.1574-6976.2009.00198.x 20030731

[25] Guenther S Ewers C Wieler LH. Extended-spectrum beta-lactamases producing *E. coli* in wildlife, yet another form of environmental pollution? Front Microbiol. (2011) 2:246. 10.3389/fmicb.2011.00246 22203818 PMC3244693

[26] Wang J Gibbons JF Mcgrath K Bai L Li F Leonard F. Molecular characterization of bla ESBL-producing *Escherichia coli* cultured from pig farms in Ireland. J Antimicrob Chemother. (2016) 71:3062–5. 10.1093/jac/dkw278 27494914

[27] CLSI. Performance Standards for Antimicrobial Disk and Dilution Susceptibility Tests for Bacteria Isolated from Animals. CLSI Document M31-A3. Wayne: Clinical and Laboratory Standards Institute (2008).

[28] CLSI. Performance Standards for Antimicrobial Susceptibility Testing. Nineteenth Informational Supplement; CLSI Document M100-S19. Wayne: Clinical and Laboratory Standards Institute (2009).

[29] CLSI. Performance Standards for Antimicrobial Susceptibility Testing. Twenty-Second Informational Supplement; CLSI Document M100-S22. Wayne: Clinical and Laboratory Standards Institute (2011).

[30] CLSI. Performance Standards for Antimicrobial Susceptibility Testing. Twenty-Fifth Informational Supplement; CLSI Document M100-S25. Wayne: Clinical and Laboratory Standards Institute (2015).

[31] Stegemann MR Passmore CA Sherington J Lindeman CJ Papp G Weigel DJ. Antimicrobial activity and spectrum of cefovecin, a new extended- spectrum cephalosporin, against pathogens collected from dogs and cats in Europe and North America. Antimicrob Agents Chemother. (2006) 50:2286–92. 10.1128/AAC.00077-06 16801403 PMC1489759

[32] Dallenne C Da Costa A Decré D Favier C Arlet G. Development of a set of multiplex PCR assays for the detection of genes encoding important β-lactamases in Enterobacteriaceae. J Antimicrob Chemother. (2010) 65:490–5. 10.1093/jac/dkp498 20071363

[33] Clermont O Christenson JK Denamur E Gordon DM. The Clermont *Escherichia coli* phylo-typing method revisited: improvement of specificity and detection of new phylo-groups. Environ Microbiol Rep. (2013) 5:58–65. 10.1111/1758-2229.12019 23757131

[34] Lescat M Clermont O Woerther PL Glodt J Dion S Skurnik D. Commensal Escherichia coli strains in Guiana reveal a high genetic diversity with host-dependant population structure. Environ Microbiol Rep. (2013) 5:49–57. 23757130 10.1111/j.1758-2229.2012.00374.x

[35] Clermont O Lescat M O'Brien CL Gordon DM Tenaillon O Denamur E. Evidence for a human-specific *Escherichia coli* clone. Environ Microbiol. (2008) 10:1000–6. 10.1111/j.1462-2920.2007.01520.x 18177373

[36] Yamamoto S Terai A Yuri K Kurazono H Takeda Y Yoshida O. Detection of urovirulence factors in *Escherichia coli* by multiplex polymerase chain reaction. FEMS Immunol Med Microbiol. (1995) 12:85–90. 10.1111/j.1574-695X.1995.tb00179.x 8589667

[37] Gérardin J Lalioui L Jacquemin E Le Bouguénec C Mainil JG. The afa-related gene cluster in necrotoxigenic and other *Escherichia coli* from animals belongs to the afa-8 variant. Vet Microbiol. (2000) 76:175–84. 10.1016/S0378-1135(00)00234-0 10946147

[38] Johnson JR Stell AL. Extended virulence genotypes of *Escherichia coli* strains from patients with urosepsis in relation to phylogeny and host compromise. J Infect Dis. (2000) 181:261–72. 10.1086/315217 10608775

[39] Tramuta C Robino P Nucera D Salvarani S Banche G Malabaila A. Molecular characterization and antimicrobial resistance of faecal and urinary *Escherichia coli* isolated from dogs and humans in Italy. Vet Ital. (2014) 50:23–30. 10.12834/VetIt.1304.09 24715590

[40] Liu X Liu H Li Y Hao C. Association between virulence profile and fluoroquinolone resistance in *Escherichia coli* isolated from dogs and cats in China. J Infect Dev Ctries. (2017) 11:306–13. 10.3855/jidc.8583 28459221

[41] Chen L Zheng D Liu B Yang J Jin Q. VFDB 2016: Hierarchical and refined dataset for big data analysis−10 years on. Nucleic Acids Res. (2016) 44:D694–7. 10.1093/nar/gkv1239 26578559 PMC4702877

[42] Marchesi JR Sato T Weightman AJ Martin TA Fry JC Hiom SJ. Design and evaluation of useful bacterium-specific PCR primers that amplify genes coding for bacterial 16S rRNA. Appl Environ Microbiol. (1998) 64:795–9. 10.1128/AEM.64.2.795-799.1998 9464425 PMC106123

[43] Jia B Raphenya AR Alcock B Alcock B Waglechner N Guo P. CARD 2017: Expansion and model-centric curation of the comprehensive antibiotic resistance database. Nucleic Acids Res. (2017) 45:D566–73. 10.1093/nar/gkw1004 27789705 PMC5210516

[44] Magiorakos AP Srinivasan A Carey RB Carmeli Y Falagas ME Giske CG. Multidrug-resistant, extensively drug-resistant and pandrug-resistant bacteria: an international expert proposal for interim standard definitions for acquired resistance. Clin Microbiol Infect. (2011) 18:268–81. 10.1111/j.1469-0691.2011.03570.x 21793988

[45] Morro M Beran G Hoffman L Griffith R. Effects of cold stress on the antimicrobial drug resistance of *Escherichia coli* of the intestinal flora of swine. Lett Appl Microbiol. (1998) 27:251–4. 10.1046/j.1472-765X.1998.t01-13-00449.x 9830139

[46] Peterson G Kumar A Gart E Narayanan S. Catecholamines increase conjugative gene transfer between enteric bacteria. Microb Pathog. (2011) 51:1–8. 10.1016/j.micpath.2011.03.002 21419838

[47] Verbrugghe E Boyen F Gaastra W Bekhuis L Leyman B Van Parys A. The complex interplay between stress and bacterial infections in animals. Vet Microbiol. (2012) 155:115–27. 10.1016/j.vetmic.2011.09.012 21963418

[48] Yildiz S Mazel-Sanchez B Kandasamy M Manicassamy B Schmolke M. Influenza A virus infection impacts systemic microbiota dynamics and causes quantitative enteric dysbiosis. Microbiome. (2018) 6:1–17. 10.1186/s40168-017-0386-z 29321057 PMC5763955

[49] Yuan L Hensley C Mahsoub H Ramesh A Zhou P. Microbiota in viral infection and disease in humans and farm animals. Prog Mol Biol Transl Sci. (2020) 171:15–60. 10.1016/bs.pmbts.2020.04.005 32475521 PMC7181997

[50] Morris D O'Hare C Glennon M Maher M Corbett-Feeney G Cormican M. Extended-spectrum beta-lactamases in Ireland, including a novel enzyme, TEM-102. Antimicrob Agents Chemother. (2003) 47:2572–8. 10.1128/AAC.47.8.2572-2578.2003 12878521 PMC166109

[51] Galvin S Boyle F Hickey P Vellinga A Morris D Cormican M. Enumeration and characterization of antimicrobial-resistant escherichia coli bacteria in effluent from municipal, hospital, and secondary treatment facility sources. Appl Environ Microbiol. (2010) 76:4772–9. 10.1128/AEM.02898-09 20525867 PMC2901727

[52] Karczmarczyk M Abbott Y Walsh C Leonard N Fanning S. Characterization of multidrug-resistant *Escherichia coli* isolates from animals presenting at a University Veterinary Hospital. Appl Environ Microbiol. (2011) 77:7104–12. 10.1128/AEM.00599-11 21856835 PMC3194860

[53] Burke L Humphreys H Fitzgerald-Hughes D. The revolving door between hospital and community: extended-spectrum beta-lactamase-producing *Escherichia coli* in Dublin. J Hosp Infect. (2012) 81:192–8. 10.1016/j.jhin.2012.04.021 22658893

[54] Burke L Humphreys H Fitzgerald-Hughes D. The molecular epidemiology of resistance in cefotaximase-producing *Escherichia coli* clinical isolates from Dublin, Ireland. Microb Drug Resist. (2016) 22:552–8. 10.1089/mdr.2015.0154 27003161

[55] Carroll D Wang J Fanning S McMahon BJ. Antimicrobial resistance in wildlife: implications for public health. Zoonoses Public Health. (2015) 62:534–42. 10.1111/zph.12182 25639901

[56] Stedt J Bonnedahl J Hernandez J Waldenström J McMahon BJ Tolf C. Carriage of CTX-M type extended spectrum β-lactamases (ESBLs) in gulls across Europe. Acta Vet Scand. (2015) 57:74. 10.1186/s13028-015-0166-3 26526188 PMC4629291

[57] Leimbach A Hacker J Dobrindt U. *E. coli* as an all-rounder: the thin line between commensalism and pathogenicity. Curr Top Microbiol Immunol. (2013) 358:3–32. 10.1007/82_2012_303 23340801

[58] O'Donnell PM Aviles H Lyte M Sonnenfeld G. Enhancement of *in vitro* growth of pathogenic bacteria by norepinephrine : importance of inoculum density and role of transferrin. Appl Environ Microbiol. (2006) 72:5097–9. 10.1128/AEM.00075-06 16820514 PMC1489335

[59] Freestone PPE Sandrini SM Haigh RD Lyte M. Microbial endocrinology: how stress influences susceptibility to infection. Trends Microbiol. (2007) 16:55–64. 10.1016/j.tim.2007.11.005 18191570

[60] Lyte M Freestone PPE. Microbial Endocrinology: Interkingdom Signaling in Infectious Disease and Health. New York, NY: Springer-Verlag (2010).

[61] Lyte M. Microbial endocrinology in the pathogenesis of infectious disease. Microbiol Spectr. (2016) 4:1–24. 10.1128/microbiolspec.VMBF-0021-2015 27227308

[62] Zhao N Wang S Li H Liu S Meng L Luo M. Influence of novel highly pathogenic avian influenza A (H5N1) virus infection on migrating whooper swans fecal microbiota. Front Cell Infect Microbiol. (2018) 8:46. 10.3389/fcimb.2018.00046 29520341 PMC5827414

[63] Gordon DM Cowling A. The distribution and genetic structure of *Escherichia coli* in Australian vertebrates: host and geographic effects. Microbiology. (2003) 149:3575–86. 10.1099/mic.0.26486-0 14663089

[64] Tenaillon O Skurnik D Picard B Denamur E. The population genetics of commensal *Escherichia coli*. Nat Rev Microbiol. (2010) 8:207–17. 10.1038/nrmicro2298 20157339

[65] Keren Y Keshet D Eidelman M Geffen M Raz-Pasteur A Hussein K. Is leclercia adecarboxylata a new and unfamiliar marine pathogen? J Clin Microbiol. (2014) 52:1775–6. 10.1128/JCM.03239-13 24523466 PMC3993675

[66] Mazzariol A Zuliani J Fontana R Cornaglia G. Isolation from blood culture of a leclercia adecarboxylata strain producing an SHV-12 extended-spectrum beta-lactamase. J Clin Microbiol. (2003) 41:1738–9. 10.1128/JCM.41.4.1738-1739.2003 12682173 PMC153879

[67] Stock I Burak S Wiedemann B. Natural antimicrobial susceptibility patterns and biochemical profiles of Leclercia decarboxylata strains. Clin Microbiol Infect. (2004) 10:724–33. 10.1111/j.1469-0691.2004.00892.x 15301675

[68] Cruz AT Cazacu AC Allen CH. Pantoea agglomerans, a plant pathogen causing human disease. J Clin Microbiol. (2007) 45:1989–92. 10.1128/JCM.00632-07 17442803 PMC1933083

[69] Deletoile A Decre D Courant S Passet V Audo J Grimont P. Phylogeny and identification of pantoea species and typing of pantoea agglomerans strains by multilocus gene sequencing. J Clin Microbiol. (2009) 47:300–10. 10.1128/JCM.01916-08 19052179 PMC2643697

[70] Khajuria A Praharaj AK Grover N Kumar M. First report of an enterobacter ludwigii isolate coharboring NDM-1. Antimicrob Agents Chemother. (2013) 57:5189–90. 10.1128/AAC.00789-13 23877704 PMC3811430

[71] Flores-Carrero A Labrador I Paniz-Mondolfi A Peaper DR Towle D Araque M. Nosocomial outbreak of extended-spectrum β-lactamase-producing *Enterobacter ludwigii* co-harbouring CTX-M-8, SHV-12 and TEM-15 in a neonatal intensive care unit in Venezuela. J Glob Antimicrob Resist. (2016) 7:114–8. 10.1016/j.jgar.2016.08.006 27750157

[72] Literak I Dolejska M Radimersky T Klimes J Friedman M Aarestrup FM. Antimicrobial-resistant faecal *Escherichia coli* in wild mammals in central Europe: multiresistant *Escherichia coli* producing extended-spectrum beta-lactamases in wild boars. J Appl Microbiol. (2009) 108:1702–11. 10.1111/j.1365-2672.2009.04572.x 19849769

[73] Dolejska M Duskova E Rybarikova J Janoszowska D Roubalova E Dibdakova K. Plasmids carrying blaCTX-M-1 and qnr genes in *Escherichia coli* isolates from an equine clinic and a horseback riding centre. J Antimicrob Chemother. (2011) 66:757–64. 10.1093/jac/dkq500 21393204

